# Minocycline Treatment Improves Memory and Reduces Anxiety by Lowering Levels of Brain Amyloid Precursor Protein and Indoleamine 2,3-Dioxygenase in a Rat Model of Streptozotocin-Induced Alzheimer’s Disease

**DOI:** 10.3390/ijms26199397

**Published:** 2025-09-26

**Authors:** Grzegorz Świątek, Jowita Nowakowska-Gołacka, Monika Słomińska-Wojewódzka, Wojciech Glac, Oliwia Harackiewicz, Ewelina Kurowska-Rucińska, Danuta Wrona

**Affiliations:** 1 Department of Animal and Human Physiology, Faculty of Biology, University of Gdansk, 59 Wita Stwosza Str, 80-308 Gdansk, Poland; grzegorz.swiatek@phdstud.ug.edu.pl (G.Ś.); wojciech.glac@ug.edu.pl (W.G.); oliwia.harackiewicz@phdstud.ug.edu.pl (O.H.); ewelina.kurowska@ug.edu.pl (E.K.-R.); 2 Department of Medical Biology and Genetics, Faculty of Biology, University of Gdansk, 59 Wita Stwosza Str, 80-308 Gdansk, Poland; jowita.nowakowska@gmail.com (J.N.-G.); monika.slominska-wojewodzka@ug.edu.pl (M.S.-W.)

**Keywords:** minocycline, memory, anxiety, amyloid precursor protein, indoleamine 2,3-dioxygenase, inflammation, sporadic Alzheimer’s disease

## Abstract

Minocycline (MINO), a classic antibiotic, may have psychotropic activity related to the modulation of the tryptophan-kynurenine pathway. In this study, we investigated the effects of MINO on (1) memory and anxiety behaviors, (2) the modulation of brain levels of amyloid precursor protein (APP) and 2,3-indoleamine dioxygenase (IDO1) levels, and (3) peripheral inflammatory markers in a streptozotocin (STZ)-induced rat model of sporadic Alzheimer’s disease (sAD). After repeated treatment with a dose of 35 mg/kg MINO for seven consecutive days, male Wistar rats with sAD showed (1) improvements in early (29 days after injection, probe test) reference memory (decreased latency to reach the platform, increased time in the critical quadrant of the Morris water maze) and anxiety disorders (increased time in the open arms of the elevated plus maze; increased exploration and entrances in the center of the white–light illuminated open field) 45–46 and 90–91 days after STZ injection; (2) reduced APP and IDO1 levels in the hippocampus and prefrontal cortex; and (3) induction of anti-inflammatory response in blood (increased TCD4^+^ lymphocyte number and interleukin-10 production). This suggests that MINO, due to its anti-inflammatory action, improves memory and anxiety behavior related to sAD, indicating its neuroprotective and psychotropic properties.

## 1. Introduction

Dysregulation of the tryptophan-kynurenine (TKP) pathway is widely implicated in pathophysiology of neurodegenerative diseases such as Alzheimer’s disease (AD) and psychiatric complaints, including depression and anxiety disorders [1,2]. Indoleamine 2,3-dioxygenase (IDO1)-related kynurenine metabolism occurring in glial cells have been reported to play a prominent role in the processes of neuroinflammation and depressive-like behavior in a model of sporadic AD (sAD) [3,4,5]. The IDO1 enzyme that catalyzes tryptophan (l-Trp) degradation together with the kynurenine pathway [6] is induced by pro-inflammatory cytokines during chronic inflammation, e.g., in the case of aging and age-associated diseases [7,8,9,10]. In the brain, IDO1 can be formed in microglia under the influence of interferon-γ (IFN-γ) produced by CD4^+^ helper T lymphocytes triggering a negative feedback loop that results in reduced neuroinflammation. However, this protective effect may be counterbalanced through the production of neurotoxic TKP metabolites such as quinolinic acid, which is formed following induction of IDO1 which, in turn, elicits oxidative stress and leads to neuronal apoptosis [7]. Loss of l-Trp suppresses immune cell proliferation in affected tissues, but in addition decreases serotonin production, which predisposes the patient to mental disorders [11,12,13].

A number of studies have demonstrated the presence of markers of inflammation in the brains of patients with AD, involving elevated levels of cytokines, chemokines, and microglia in affected regions [14]. Pro-inflammatory cytokines have been linked to increased tau phosphorylation and decreased synaptophysin levels, indicating their role in cytoskeletal and synaptic changes that occur in AD [15]. Amyloid β (Aβ) peptide performs a physiological role in synaptic plasticity and neuronal survival, but an imbalance between its production and clearance facilitates its accumulation and subsequent toxicity and may lead to neuroinflammation through the activation of the innate immune system [15,16,17]. AD’s amyloidogenic pathology is characterized by extracellular senile plaques caused by the aggregation of insoluble amyloid fibrils which are involved in neuroinflammation and neurodegeneration [16,18,19,20,21,22]. Aβ peptide is a 4-kDa peptide that results from the proteolytic cleavage of the transmembrane amyloid precursor protein (APP) [20]. The amyloidogenic pathway, in which APP is cleaved at many sites, effectively by β-secretase, BACE-1 (β-site APP-cleaving enzyme 1), and the γ-secretase complex formed by presenilins, leading to the formation of Aβ peptides of varying lengths, such as Aβ40 and Aβ42 [23,24]. Nevertheless, while early-onset AD results from genetic overproduction of Aβ [25], sporadic AD (sAD) is more likely the consequence of impaired Aβ clearance [26]. The formation of senile plaques is mediated by changes in APP metabolism, where enzymes such as β- and γ-secretases augment the formation of amyloid peptides that are more prone to forming insoluble aggregates and accumulating in the brain with sAD [27].

Minocycline (MINO), a classic antibiotic, can produce psychotropic activity due to modulation of TKP, including IDO1 [1,2]. MINO is a tetracycline derivative that integrates anti-inflammatory activities with neuroprotective abilities by reducing inflammation and oxidative stress [28,29]. Anti-inflammatory activities of MINO include inhibition of nitric oxide synthase [30] and upregulation of interleukin 10 [31]. MINO can efficiently cross the blood–brain barrier (BBB) because of its small size (495 d) and lipophilic properties [32], and it is been shown to have neuroprotective activity that is different from its bacteriostatic action in animal models of cerebral ischemia [33,34], Parkinson’s disease, and Huntington’s disease [35]. The anti-inflammatory, antioxidant and anti-apoptotic activities of MINO account for the resurgence of interest in its use as an adjuvant treatment in psychiatric and neurological disorders [36].

Neuroinflammation and depressive/anxiety symptoms are well-established indicators in the clinical and preclinical phenotype of AD and there is a lack of information on anxiety related to sAD. We investigated in this study the potential protective effect of MINO short term treatment (35 mg/kg once daily intraperitoneal injections for seven consecutive days) on early, pre-plaque stages of the amyloid pathology, which should correspond to the earliest, pre-clinical stages in the human and late stage of memory impairment and anxiety-like behavior in a rat model of sAD induced by intracerebroventricular (ICV) administration of streptozotocin (STZ).

In this neuroinflammation model of sAD, insulin resistance in the brain, which is an early symptom of sAD [37,38], induces the activation of pro-inflammatory, pro-apoptotic, and pro-APP-Aβ cascades [39] and influences the expression and transformation of the tau protein [40] by favoring oxidative stress, reactivating oxygen forms, mitochondrial dysfunction, and DNA damage. All these actions are involved in neurodegeneration [41,42,43]. In addition, in the ICV-STZ model, IDO1 induction in response to upregulation of innate immune and pro-inflammatory cytokines was involved in the regulation of sAD-associated depression [44,45,46].

Our results indicate that MINO alleviates STZ-induced early reference memory deficits and anxiety disorders at the late stage of sAD progression. These neuroprotective effects of MINO were associated with reduced brain APP and IDO1 levels and induction of peripheral anti-inflammatory response. This suggests that the antibiotic MINO shows potential as a therapeutic for sAD treatment and anxiety disorders, in particular, because of its anti-inflammatory features.

## 2. Results

### 2.1. Behavioral Activity

#### 2.1.1. Reference Memory Performance in the Probe Test on Day 4 of the Morris Water Maze (MWM) Test

**Figure 1:**
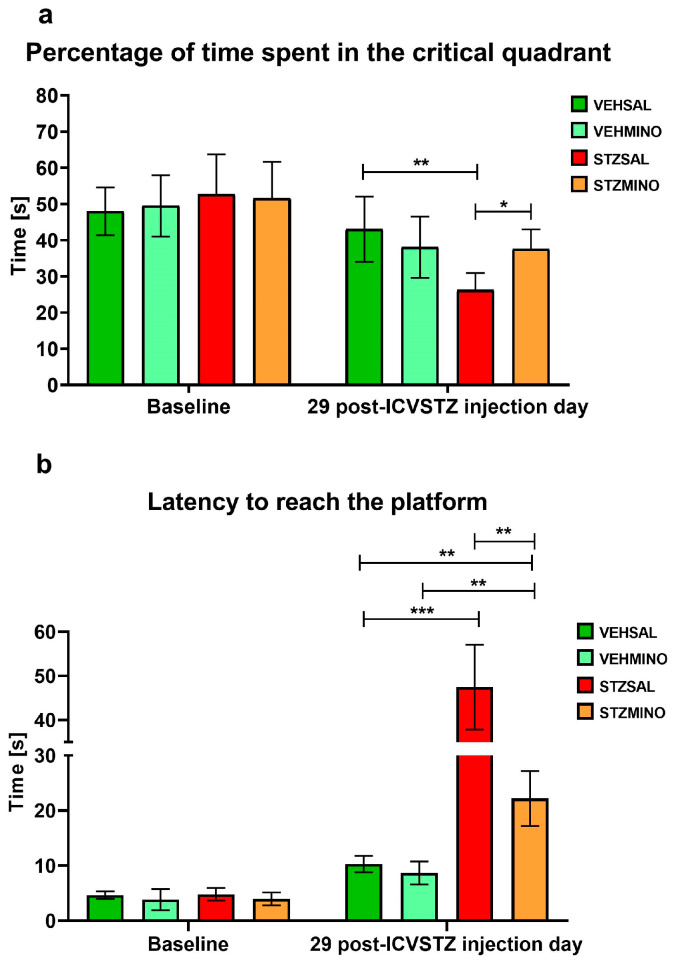
Reference memory behavior in the course of the probe test on the 4th day of the Morris water maze (MWM) test, assessed as percentage of time spent in the critical quadrant (**a**) and latency to reach the place where the platform used to be (**b**), in rats prior to cannula implantation and injections (BASELINE, *n* = 40) and at day 29 after intracerebroventricular (ICV) injection of streptozotocin (STZ) or citrate buffer (VEH) and intraperitoneal (i.p.) injection of saline (SAL) for 7 consecutive days (STZSAL, *n* = 10; VEHSAL, *n* = 10) or ICV injection of STZ or VEH and i.p. minocycline (MINO) injection for 7 consecutive days (STZMINO, *n* = 10; VEHMINO, *n* = 10). Explanations: Data are presented as mean ± SD and were analyzed using Mann–Whitney U test; asterisks over the lines between the bars show the significance between the two groups, *—*p* < 0.05, **—*p* < 0.01, ***—*p* < 0.001.

Reference memory during the probe test is presented in Figure 1 and shows reference memory performance during a single trial on day 4 of the MWM when the platform was removed.

As shown in Figure 1a, there was a significant decrease in the percentage of time spent in the critical quadrant in the STZSAL animals compared to the control VEHSAL group (*p* < 0.01) and rats with STZ injection and treated with minocycline (STZMINO, *p* < 0.05). Moreover, rats from the STZMINO group spent similar time in the critical quadrant compared to both control groups (VEHSAL and VEHMINO). In the STZSAL rats, latency to reach the platform was significantly longer than in the STZMINO group (*p* < 0.01) and control VEHSAL and VEHMINO animals (Figure 1b, *p* < 0.01). On the other hand, there was significantly longer latency to reach the platform in the STZMINO group compared to both control groups (*p* < 0.01).

#### 2.1.2. Anxiety Behavior in the Elevated Plus Maze (EPM) Test

**Figure 2:**
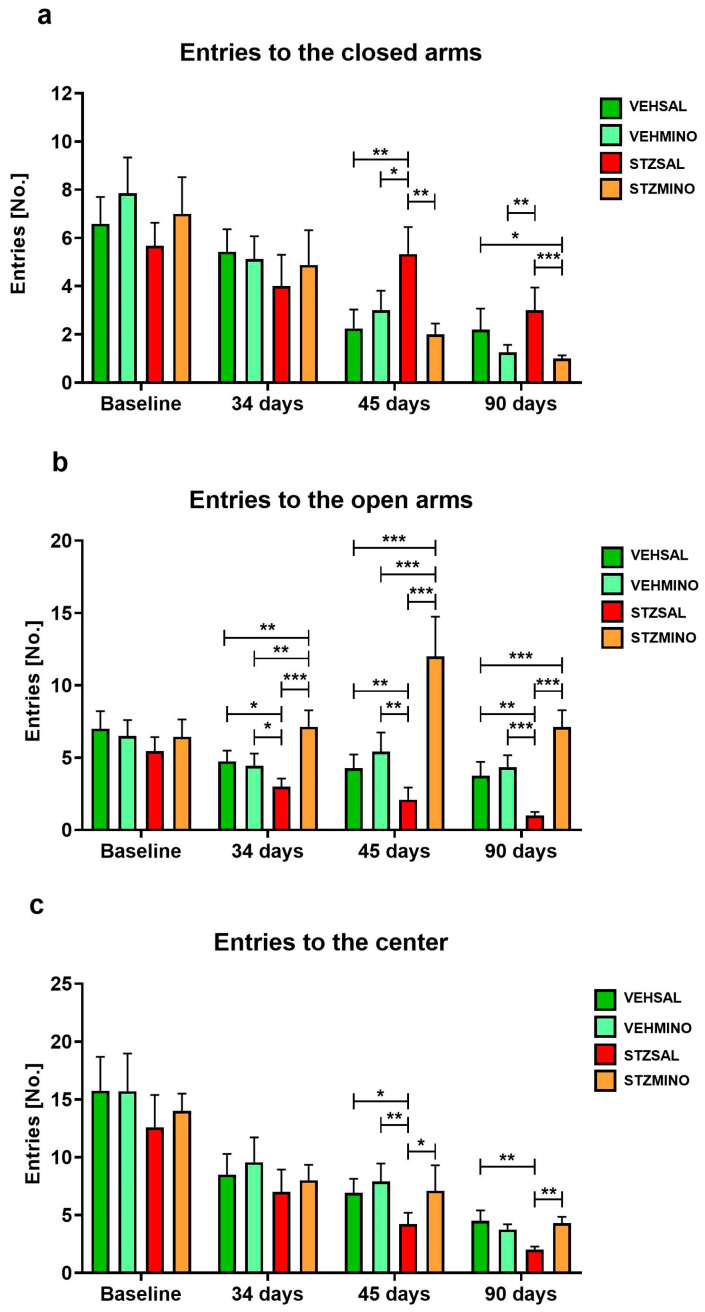
Anxiety behavior presented as entries to the closed arms (**a**), entries to the open arms (**b**), and entries to the center (**c**) of the elevated plus maze (EPM) test in rats prior to cannula implantation and injections (BASELINE, *n* = 40) and at days 34, 45, and 90 after intracerebroventricular (ICV) injection of streptozotocin (STZ) or citrate buffer (VEH) and intraperitoneal (i.p.) injection of saline (SAL) for 7 consecutive days (STZSAL, *n* = 10; VEHSAL, *n* = 10) or ICV injection of STZ or VEH and i.p. minocycline (MINO) injection for 7 consecutive days (STZMINO, *n* = 10; VEHMINO, *n* = 10). Explanations: Data are presented as mean ± SD and were analyzed using Mann–Whitney U test; asterisks over the lines between the bars show the significance between the two groups, *—*p* < 0.05, **—*p* < 0.01, ***—*p* < 0.001.

**Figure 3:**
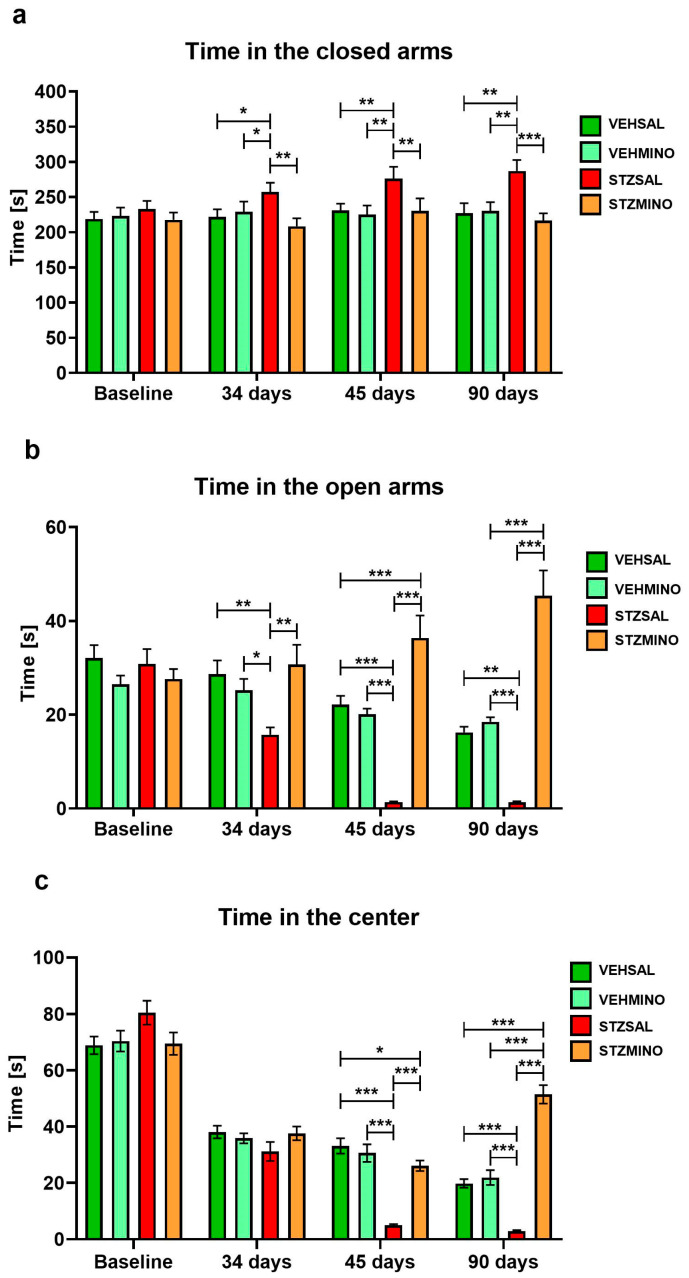
Anxiety behavior presented as time spent in the closed arms (**a**), time spent in the open arms (**b**), and time spent in the center (**c**) of the elevated plus maze (EPM) test in rats prior to cannula implantation and injections (BASELINE, *n* = 40) and at days 34, 45, and 90 after intracerebroventricular (ICV) injection of streptozotocin (STZ) or citrate buffer (VEH) and intraperitoneal (i.p.) injection of saline (SAL) for 7 consecutive days (STZSAL, *n* = 10; VEHSAL, *n* = 10) or ICV injection of STZ or VEH and i.p. minocycline (MINO) injection for 7 consecutive days (STZMINO, *n* = 10; VEHMINO, *n* = 10). Explanations: Data are presented as mean ± SD and were analyzed using Mann–Whitney U test; asterisks over the lines between the bars show the significance between the two groups, *—*p* < 0.05, **—*p* < 0.01, ***—*p* < 0.001.

Figure 2 and Figure 3 present behavioral activity in the elevated plus maze test (EPM) associated with anxiety, measured as frequency of entries to the closed arms (Figure 2a), to the open arms (Figure 2b), and center of the maze (Figure 2c) as well as time spent in these parts of the maze (Figure 3a, Figure 3b, and Figure 3c, respectively).

Rats from the STZMINO group more frequently (in all comparisons *p* < 0.001) entered open arms of the maze compared to the STZSAL and control rats 34, 45, and 90 days after ICVSTZ administration (Figure 2b). Furthermore, MINO treatment of the rats with the sAD model significantly increased the frequency of entries to the center of the labyrinth compared to STZSAL animals 45 (*p* < 0.05) and 90 (*p* < 0.01) days after ICVSTZ injection (Figure 2c). Compared to the control groups, a significantly reduced frequency of entries into the open arms of the maze was observed in STZSAL rats at 45 (in all comparisons *p* < 0.01) and 90 (*p* < 0.01 vs. VEHSAL, *p* < 0.001 vs. VEHMINO) days after STZ administration. There was also a significant reduction in entries in the center of the EPM in STZSAL rats compared to the control VEHSAL (45 days, *p* < 0.05; 90 days, *p* < 0.01) and VEHMINO (45 days, *p* < 0.01) animals.

The STZMINO group spent more time in the open arms compared to the STZSAL rats at 34 days (*p* < 0.01), 45 days (*p* < 0.001), and 90 days (*p* < 0.001) after ICVSTZ administration (Figure 3b). In addition, time spent in the open arms was longer in rats with the sAD model and MINO treatment compared to the control VEHSAL group at 45 days (*p* < 0.001) and to VEHMINO animals at 90 days (*p* < 0.001) after ICVSTZ injection. At 45 and 90 days after ICVSTZ injection, rats treated with MINO spent more time in the center of the maze than rats in the STZSAL group (Figure 3c, *p* < 0.001 in all comparisons). Time spent in the center was longer in the STZMINO group compared to VEHSAL and VEHMINO (*p* < 0.001 in both comparisons) at 90 days after sAD induction. Rats from STZSAL group spent significantly less time in the center of the maze than control VEHSAL and VEHMINO at 45 and 90 days after STZ injection (*p* < 0.001 in all comparisons).

#### 2.1.3. Anxiety Behavior in the White and Light Illuminated Open Field (OF) Test

**Figure 4:**
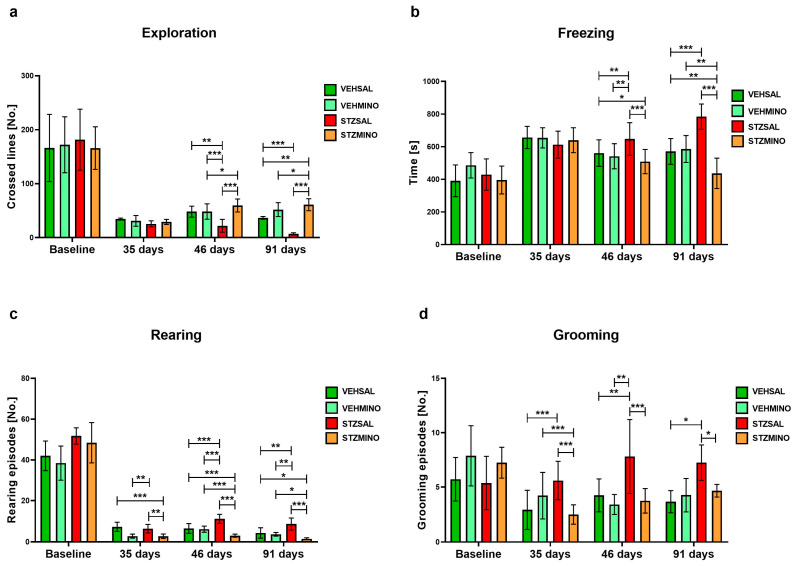
Anxiety behavior presented as exploration (**a**), freezing (**b**), number of rearing (**c**), and number of grooming (**d**) in the white and light illuminated open field (OF) test in rats prior to cannula implantation and injections (BASELINE, *n* = 40) and at days 35, 46, and 91 after intracerebroventricular (ICV) injection of streptozotocin (STZ) or citrate buffer (VEH) and intraperitoneal (i.p.) injection of saline (SAL) for 7 consecutive days (STZSAL, *n* = 10; VEHSAL, *n* = 10) or ICV injection of STZ or VEH and i.p. minocycline (MINO) injection for 7 consecutive days (STZMINO, *n* = 10; VEHMINO, *n* = 10). Explanations: Data are presented as mean ± SD and were analyzed using Mann–Whitney U test; asterisks over the lines between the bars show the significance between the two groups, *—*p* < 0.05, **—*p* < 0.01, ***—*p* < 0.001.

**Figure 5:**
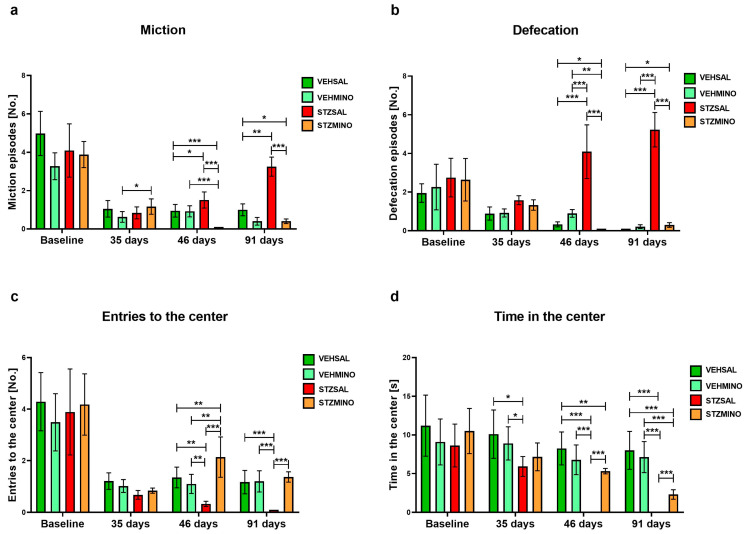
Anxiety behavior presented as miction (**a**), defecation (**b**), entries to the center (**c**), and time in the center (**d**) in the white and light illuminated open field (OF) test in rats prior to cannula implantation and injections (BASELINE, *n* = 40) and at days 35, 46, and 91 after intracerebroventricular (ICV) injection of streptozotocin (STZ) or citrate buffer (VEH) and intraperitoneal (i.p.) injection of saline (SAL) for 7 consecutive days (STZSAL, *n* = 10; VEHSAL, *n* = 10) or ICV injection of STZ or VEH and i.p. minocycline (MINO) injection for 7 consecutive days (STZMINO, *n* = 10; VEHMINO, *n* = 10). Explanations: Data are presented as mean ± SD and were analyzed using Mann–Whitney U test; asterisks over the lines between the bars show the significance between the two groups, *—*p* < 0.05, **—*p* < 0.01, ***—*p* < 0.001.

Figure 4 and Figure 5 show behavioral activity related to anxiety in the white, light illuminated open field (OF) test measured as exploration (Figure 4a), freezing (Figure 4b), rearing (Figure 4c), grooming (Figure 4d), miction (Figure 5a), defecation (Figure 5b), entries to the center (Figure 5c), and time spent in the center of the maze (Figure 5d).

As shown in Figure 4, exploration as indicated by the number of lines crossed was significantly reduced in the STZSAL rats compared to the STZMINO-treated animals at days 46 and 91 (in both comparisons *p* < 0.001) after ICVSTZ injection. Furthermore, exploration was significantly lower in the STZSAL group, while it was significantly higher in the STZMINO rats compared to the controls at 46 and 91 days after sAD induction. Time of freezing was significantly shorter in the rats with sAD model treated with MINO compared to the rats from the STZSAL group at 46 and 91 days after ICVSTZ injection (in both comparisons *p* < 0.001). The STZMINO rats also showed shorter time of freezing than the control VEHSAL and VEHMINO rats, in particular at 91 days after sAD induction (in both comparisons *p* < 0.01). On the other hand, there was a significantly longer time of freezing in the STZSAL rats than in VEHSAL and VEHMINO control groups (46 days: in both comparisons *p* < 0.01); 91 days: *p* < 0.001). Furthermore, number of rearing (Figure 4c) and grooming (Figure 4d) episodes was significantly higher in the STZSAL animals compared to the STZMINO rats at days 35 (rearing, *p* < 0.01; grooming *p* < 0.001), 46 (rearing and grooming in both comparisons *p* < 0.001), and 91 (rearing, *p* < 0.001, grooming *p* < 0.05). At days 45 and 91 days after ICVSTZ injection, the lower number of miction and defecation episodes in the STZMINO rats than in STZSAL animals (45 and 91 days in all comparisons *p* < 0.001) was observed (Figure 5a,b). Furthermore, rats from the STZMINO group more frequently entered the center of the maze and spent more time in the center compared to the STZSAL group at days 45 (Figure 5c) and 90 (Figure 5d, in all comparisons *p* < 0.001).

### 2.2. Plasma Cytokine Concentration and Production, and TCD4^+^/TCD8^+^ Lymphocyte Number

**Figure 6:**
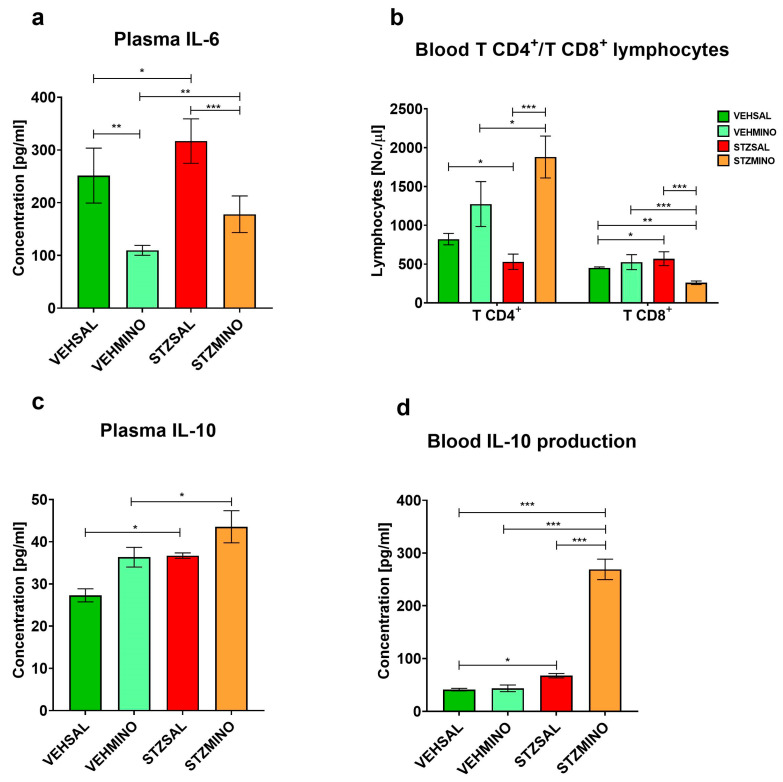
Concentration of plasma interleukin (IL)-6 (IL-6) (**a**), IL-10 (**c**), number of blood TCD4^+^ and TCD8^+^ lymphocytes (**b**), and concanavalin-A (Con-A)-stimulated blood IL-10 production (**d**) in rats at 47 day after intracerebroventricular (ICV) injection of streptozotocin (STZ) or citrate buffer (VEH) and intraperitoneal (i.p.) injection of saline (SAL) for 7 consecutive days (STZSAL, *n* = 10; VEHSAL, *n* = 10) or ICV injection of STZ or VEH and i.p. minocycline (MINO) injection for 7 consecutive days (STZMINO, *n* = 10; VEHMINO, *n* = 10). Explanations: Data are presented as mean ± SD and were analyzed using Mann–Whitney U test; asterisks over the lines between the bars show the significance between the two groups, *—*p* < 0.05, **—*p* < 0.01, ***—*p* < 0.001.

Plasma concentration of interleukin (IL)-6, IL-10, number of peripheral blood TCD4^+^ and TCD8^+^ lymphocyte number, and concanavalin-A (Con-A)-stimulated production of IL-10 are presented in Figure 6.

Compared to the control VEHSAL (*p* < 0.05) and the STZMINO (*p* < 0.001) rats, plasma concentration of IL-6 significantly increased in the STZSAL group 47 days after ICVSTZ administration (Figure 6a). On the other hand, treatment of rats with the sAD model with MINO resulted in a significant increase in plasma IL-10 concentration compared to the VEHSAL control group (*p* < 0.05) and an increase in Con-A-stimulated IL-10 production in the peripheral blood compared to the controls and STZSAL rats (in all comparisons *p* < 0.001, Figure 6c and Figure 6d, respectively). In the STZMINO group, the ability of PBMC T cells to produce Con-A-stimulated IL-6 was inhibited, as indicated by the lack of a significant difference in the concentration of this pro-inflammatory cytokine between Con-A-stimulated samples and corresponding control samples without Con-A stimulation (Table S1). Furthermore, there was a significant increase in the number of blood TCD4^+^ lymphocytes after ICVSTZ injection and MINO treatment compared to the STZSAL group (*p* < 0.001) and VEHMINO rats (*p* < 0.05, Figure 6b). The TCD8^+^ lymphocyte number was lower in the STZMINO group than in the STZSAL (*p* < 0.001), VEHMINO (*p* < 0.001), and VEHSAL ((*p* < 0.01), Figure 6b) animals.

### 2.3. Plasma Corticosterone Concentration

**Figure 7:**
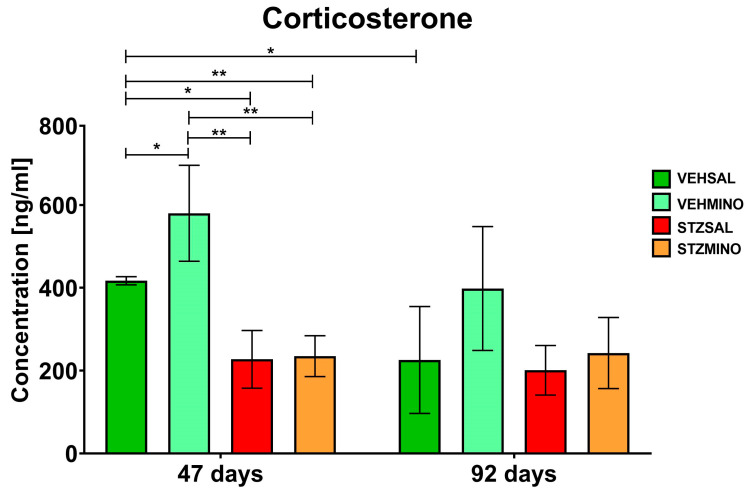
Concentration of plasma corticosterone in rats at 47 and 92 days after intracerebroventricular (ICV) injection of streptozotocin (STZ) or citrate buffer (VEH) and intraperitoneal (i.p.) injection of saline (SAL) for 7 consecutive days (STZSAL, *n* = 10; VEHSAL, *n* = 10) or ICV injection of STZ or VEH and i.p. minocycline (MINO) injection for 7 consecutive days (STZMINO, *n* = 10; VEHMINO, *n* = 10). Explanations: Data are presented as mean ± SD and were analyzed using Mann–Whitney U test; asterisks above the line between the bars show significance between the two groups, *—*p* < 0.05, **—*p* < 0.01.

Figure 7 shows plasma corticosterone concentration as a peripheral indicator of hypothalamic–pituitary–adrenal (HPA) axis activity on days 47 and 92 after ICVSTZ/SAL administration.

There were no significant differences in plasma corticosterone concentration between the STZSAL and STZMINO animals 47 and 92 days after sAD induction (Figure 7). However, compared to the controls, corticosterone concentration was significantly reduced in the STZSAL (VEHSAL, *p* < 0.05; VEHMINO, *p* < 0.01) and STZMINO (in both comparisons *p* < 0.01) groups. In addition, a significantly higher level of peripheral corticosterone in the VEHMINO (*p* < 0.05) than in the VEHSAL group was observed.

### 2.4. Amyloid Beta Precursor Protein (APP) and Indoleamine 2,3-Dioxygenase (IDO1) Protein Levels in the Hippocampus and Prefrontal Cortex

**Figure 8:**
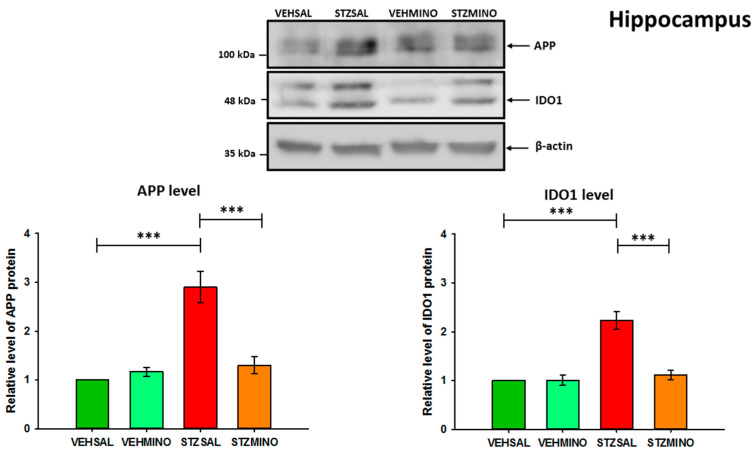
Western blot analysis of amyloid precursor protein (APP), indoleamine 2,3-dioxygenase (IDO1), and actin in the hippocampus of randomly chosen rats of each group at 92 days after intracerebroventricular (ICV) injection of streptozotocin (STZ) or citrate buffer (VEH) and intraperitoneal (i.p.) injection of saline (SAL) for 7 consecutive days (STZSAL, *n* = 5; VEHSAL, *n* = 7) or ICV injection of STZ or VEH and i.p. minocycline (MINO) injection for 7 consecutive days (STZMINO, *n* = 3; VEHMINO, *n* = 3). Explanations: The values are expressed as mean ± SD, *n* ≥ 3, *** *p* < 0.001, one-way ANOVA followed by Dunnett’s test. Molecular mass markers are shown on the left side of the blots. APP and IDO1 protein levels were quantified and shown in graphs. Values obtained for lysates of control rats (VEHSAL) were set to 1 and all other values are shown as fold changes relative to the control.

**Figure 9:**
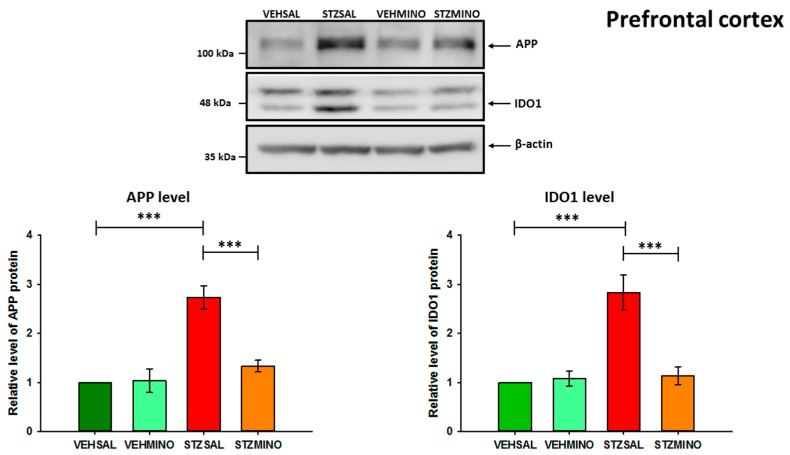
Western blot analysis of amyloid precursor protein (APP), indoleamine 2,3-dioxygenase (IDO1), and actin in the prefrontal cortex of randomly chosen rats of each group at 92 days after intracerebroventricular (ICV) injection of streptozotocin (STZ) or citrate buffer (VEH) and intraperitoneal (i.p.) injection of saline (SAL) for 7 consecutive days (STZSAL, *n* = 6; VEHSAL, *n* = 9) or ICV injection of STZ or VEH and i.p. minocycline (MINO) injection for 7 consecutive days (STZMINO, *n* = 5; VEHMINO, *n* = 5). Explanations: The values are expressed as mean ± SD, *n* ≥ 3, *** *p* < 0.001, one-way ANOVA followed by Dunnett’s test. Molecular mass markers are shown on the left side of the blots. APP and IDO1 protein levels were quantified and shown in graphs. Values obtained for lysates of control rats (VEHSAL) were set to 1 and all other values are shown as fold changes relative to the control.

The levels of APP and IDO1 proteins were analyzed by Western blotting (Figure 8, Figure 9 and Figure S1). The distinct APP bands correspond to different protein isoforms [47,48]. However, they were analyzed as total APP. For IDO1 analysis, the upper band probably represents a splicing variant, a post-translationally modified form of the protein or a non-specific product. Double bands in Western blot for IDO1 have already been observed [49,50]. Our Western blot analysis revealed a more than 2.5-fold increase in APP levels in both structures analyzed (Figure 8, Figure 9 and Figure S1). IDO1 protein levels were more than 2-fold higher in the hippocampus (Figure 8) and approximately 3-fold higher in the prefrontal cortex (Figure 9) of ICVSTZ-injected rats compared to the control VEHSAL group.

Importantly, treatment of STZ-injected rats with minocycline led to a significant reduction (*p* < 0.001) in IDO1 levels in the prefrontal cortex and hippocampus compared to STZSAL rats. These levels were similar to those observed in the control rats (VEHSAL) and minocycline-injected control animals (VEHMINO). APP levels were also significantly (*p* < 0.001) reduced in the hippocampus and prefrontal cortex of STZ/MINO rats compared to STZSAL animals (Figure 8 and Figure 9). The levels of APP were only slightly higher (not significantly) than those observed in the control groups (VEHSAL and VEHMINO).

## 3. Discussion

In this study, we have shown that seven consecutive days of MINO treatment alleviates ICVSTZ-induced deficits in early reference memory (29 days after STZ injection, probe test) and anxiety-like behavior associated with sAD in the late stage of disease progression (45–46 and 90–91 days after STZ administration) in the rat model. These neuroprotective effects of MINO were associated with reduced APP levels and IDO1 expression in the hippocampus and prefrontal cortex, as well as an increase in peripheral anti-inflammatory response, including enhanced production of anti-inflammatory IL-10.

To the best of our knowledge, our results provide the first evidence that IDO1-associated neuroinflammation in the hippocampus and prefrontal cortex may be involved in anxiety-like behaviors associated with sAD in the ICVSTZ rat model, and that MINO treatment alleviates these abnormalities. Our findings suggest that the antibiotic MINO may be a possible therapeutic drug for treating sAD and anxiety disorders based on its anti-inflammatory properties.

Studies on animal models of Alzheimer’s or Parkinson’s disease have provided evidence of the anti-inflammatory, antioxidant, anti-amyloidogenic, and anti-apoptotic effects of tetracyclines related to cognitive enhancement [51]. However, studies evaluating the effect of MINO on memory deficits induced by ICV-STZ in this neuroinflammation-based sAD model are limited. Sharma et al. [52] found that male Wistar rats treated for 17 days with MINO (10, 20, and 40 mg/kg i.p.), beginning at day one after STZ injection, revealed a remarkable dose-dependent improvement in reference memory in the MWM test. According to Vicente et al. [53], MINO treatment increased learning and memory performance and restored the sleep–wake pattern disturbed by STZ. These effects were accompanied by a reduction in microglial cell density and restoration of STZ-induced morphological changes in the locus coeruleus (LC) area and robust trend in the impact of MINO on tumor necrosis factor (TNF)-α in the LC but not IL-1β and IL-10. Our findings regarding improved reference memory during the probe test, indicated by reduced latency to reach the platform and prolonged time spent in the critical quadrant of MWM and MINO treatment (Figure 1), are consistent with these results and support the neuroprotective effect of MINO related to its anti-inflammatory properties. In an animal model of AD induced by the administration of Aβ oligomers, Mahmoudian et al. [54] demonstrated that in male Wistar rats MINO treatment (50 and 100 mg/kg/day; per os) for 30 days regained Aβ-caused learning and memory losses and prevented neuronal loss in the hippocampus and the aggregation of Aβ plaques. According to the authors, MINO neuroprotective effects on memory dysfunction were due to its antioxidant and anti-apoptotic effects. Garcez et al. [55] found that in male BALB/c mice after ICV injection of Aβ (1-42) oligomer, the administration of MINO (50 mg/kg) via oral route for 17 days reversed the memory disorders produced by Aβ (1-42). In addition, the authors observed that in the hippocampus and cortex MINO reversed rising levels of L-1β, TNF-α, and IL-10 or IL-4 due to Aβ (1-42). In other studies, the effect of MINO on spatial memory was also investigated using an AD model in rodents. Some of these studies showed that MINO was able to reverse spatial memory impairment [56,57,58]. However, another study found that it did not improve cognitive deficits [59].

Acting through several mechanisms, ranging from anti-inflammatory to antioxidant and anti-apoptotic activities, MINO is a promising drug for clinical research either for acute brain injuries or chronic neurodegenerative disorders. In an attempt to explain the mechanism of MINO’s beneficial effect on ICVSTZ-induced reference memory deficit observed on day 29 after sAD induction, we examined APP levels in the most important structures responsible for memory—the hippocampus and prefrontal cortex. We found that MINO treatment (35 mg/kg for 7 days) reduced levels of APP in both brain structures (Figure 8 and Figure 9), determined 92 days after STZ administration. This suggests that MINO prevented the generation and accumulation of Aβ in the pre-plaque phases of sAD-like amyloid-derived pathology, thereby alleviating STZ-induced memory deficits.

Similarly to our results, it has previously been shown that APP levels were markedly increased in the cortex and hippocampus of STZ-injected rats [27,60,61]. In contrast, Souza et al. [45] reported that the APP expression has not changed by ICVSTZ injection. Our findings regarding the beneficial effect of MINO on memory impairment associated with reduced APP levels in the brain in the ICVSTZ model are consistent with results obtained in other AD models. In rats with AD induced by Aβ administration, MINO treatment improved memory deficits and prevented neuronal loss in the hippocampus and Aβ plaque accumulation [54]. Furthermore, in a transgenic model of AD-related amyloid pathology [62], treatment with MINO (1 month, 50 mg/kg/day, i.p.) resulted in a reduction in inflammatory markers, which correlated with a reduction in APP levels and beta-site APP cleaving enzyme 1 (BACE-1) activity.

Depressive disturbances are the most frequent neuropsychiatric signs in Alzheimer’s disease, which affect as many as 20–40% of patients, and conventional antidepressants cannot efficiently alleviate these depressive symptoms [63], suggesting that depression related to AD may involve a different pathogenesis. There is a growing body of evidence on the role of the kynurenine pathway (KP) in the relationship between inflammation and depressive disorders [1,2,3,5,9,44,64]. However, there are few reports on the involvement of KP in the mechanisms underlying the depressive effects of ICVSTZ injection and MINO treatment in rats. There is a lack of such studies regarding the anxiolytic effects of MINO in the rat ICVSTZ model. Since ICV-STZ injections cause intense inflammation of the nervous system, which progresses to chronicity and elevates the production of pro-inflammatory and neurotoxic cytokines, particularly in the hippocampus and prefrontal cortex, leading to neuronal death [61], we then analyzed whether this neuroprotective effect of MINO on memory and anxiety disorders associated with sAD was the result of inflammation inhibition. We found that MINO-induced memory improvement and anxiolytic effects, as indicated by prolonged time spent in the open arms of the elevated plus maze, and increased exploration and number of entries into the white–light illuminated center of the open field in ICV-injected rats with STZ were associated with reduced levels of the inflammatory marker such as IDO1 in the hippocampus and prefrontal cortex (Figure 8 and Figure 9). It should be noted, however, that the control groups (VEHSAL and VEHMINO) showed, 45 and 90 days after VEH injection, a worsening of the behavioral activity of some of the parameters studied in the EPM and OF tests, compared to the anti-anxiety effect observed at that time in MINO-treated animals in the sAD model. Nevertheless, both control groups showed lower levels of anxiety than the STZSAL group in the late stage of the disease. This observation is difficult to explain and may suggest that MINO differentially affects healthy control rats (VEHMINO) and rats with STZ-induced neuroinflammation. It is possible that MINO as an antibiotic may, for example, alter the gut microbiota in inflamed and healthy animals in different ways, including *Eubacterium siraeum* and *Anaeroplasma*, which are known for their anti-inflammatory properties [65]. This differential impact of MINO may contribute to differences in anxiety behavior between controls and STZMINO animals observed in our study. However, the explanation of this effect requires further investigation.

According to our research, modulation of KP by administering MINO may be a more effective strategy for treating AD-related anxiety. Our results are consistent with those of Souza et al. [44], who demonstrated that ICV-STZ triggered IDO in the mouse hippocampus and depression-related behaviors, as quantified by prolonged immobility time in the forced swim test and reduced total time of grooming in the splash test, and MINO counteracted the development of depression-associated behavior and diminished increased regulation induced by STZ of pro-inflammatory cytokines in the hippocampus. Furthermore, MINO abolished the increase in tryptophan and kynurenine levels and protected against serotonin dysfunction in the hippocampus of mice injected with STZ. Qin et al. [46] reported that in ICV-STZ rats, IDO-related KP was activated in the prelimbic cortex and infralimbic cortex, subregions of the prefrontal cortex that perform different functions in the expression of depression and anxiety-like behaviors [66], while inhibition of IDO in these brain structures alleviated depression-like behavior in rats. According to the authors, ICV-STZ induced depression behavior before cognitive impairment in rats (already on the 7th day after ICVSTZ injections). Furthermore, short (24 h) and long (14 days) exposure to MINO at a dose of 5 mg/kg (therapeutic dosage for humans) attenuated negative impact of ICVSTZ injections on mice, including STZ-induced depressive-like behaviors and increased regulation of neuroinflammatory genes in the hippocampus [67]. The authors suggest that MINO showed protective action against acute oxidation-induced cell injuries and the resulting inflammatory reactions. In another AD model, Amari et al. [68] demonstrated that MINO therapy significantly reversed depression-like behaviors related to AD and concentrations of cytokines such as IL-10, IL-β, and TNF-α in the hippocampus of rats treated with Aβ1-42. According to the authors, the antidepressant effect of MINO may be a result of its anti-inflammatory characteristics.

It has been previously reported that T helper (CD4^+^) lymphocytes that express IFN-γ are able to stimulate microglia cells to express IDO1, which may trigger a negative feedback loop to attenuate neuroinflammation [69]. Since MINO treatment induced inhibition of neuroinflammation by reducing brain IDO1 level, we wanted to investigate whether MINO also has an anti-inflammatory effect on the peripheral immune response, including function of TCD4^+^ lymphocytes in the ICVSTZ model. In order to explain the mechanism of MINO’s action on the immune response in the blood, we aimed to measure plasma corticosterone concentrations and blood production of pro-/anti-inflammatory cytokines (IL-6/IL-10), number of peripheral blood TCD4^+^/ TCD8^+^ lymphocytes and plasma corticosterone levels. Our analysis showed that MINO treatment in ICVSTZ rats resulted in a decrease in pro-inflammatory IL-6 and an increase in anti-inflammatory IL-10, along with increased IL-10 production induced by concanavalin A (Figure 6), increased TCD4^+^ lymphocyte number, and decreased TCD8^+^ lymphocyte number. This may suggest that the main goal of MINO’s immunomodulatory action is to reduce the number and activity of Th1 cells producing Il-6 and to increase the number of Th2 cells producing IL-10. We observed similar shifts in the immune response towards anti-inflammatory effects in rats exposed to white and illuminated open field stress and antidepressant treatment with desipramine [70,71,72]. Furthermore, we did not observe significant differences in the plasma concentration of immunosuppressive hormone, such as corticosterone, between rats exposed to STZ and MINO injections and rats with the sAD model without MINO treatment on either day 47 or day 92 after ICVSTZ administration. Contrary to our findings, evidence of elevated cortisol levels in AD patients, suggesting progressive HPA axis dysregulation, which has been associated with cognitive impairment and depressive disorders, were observed [73,74]. In support, others [75] demonstrated progressive HPA axis deregulation in an acute model of AD caused by ICV injections of Aβ(25-35), which was associated with disruption of the ratio of mineralocorticoids to glucocorticoid receptors and impaired nucleocytoplasmic transport of glucocorticoid receptors. It should be noted that cortisol and anxiety levels differentially affect memory performance in patients with AD [74,76], as evidenced by a negative correlation between cortisol concentration and memory and better memory performance at moderate anxiety levels. It has recently been demonstrated that both memory and anxiety depend on synaptic plasticity in the ventral hippocampus, which contributes to the modulation of emotional responses in the early stages of AD pathology [77]. In contrast to these results, in the TgF344-AD model, no correlation between anxiety level displayed during the EPM test and memory performance in the MWM test was found [78]. However, it should be emphasized that different mechanisms underlie depression and anxiety. Dysregulated medial prefrontal cortex control over amygdale, with aberrant amygdale activation, is involved in fear and anxiety disorders [79,80,81]. Peng et al. [82] demonstrated that 7-day corticosterone treatment of male rats induced anxiety-like behavior by decreasing synaptic transmission onto the ventral tegmental area dopamine neurons which innervate brain regions that are critical for emotional processing [83] and mediate symptoms of anxiety, including the nucleus accumbens, amygdala [84], prefrontal cortex [85], and hippocampus [86]. The lack of significant differences in levels of a key marker of the HPA axis activity such as corticosterone in STZ compared to MINO groups observed in our study at late stages of sAD progression suggests that both treatments may not directly impair the functioning of HPA axis. On the other hand, corticosterone concentration was measured only twice in our study, during the late stage of AD pathology, whereas HPA axis activity may show a different response in the early compared to the late stage of the disease. It may also indicate that the anti-inflammatory properties of MINO and its beneficial effects on memory impairment and anxiety disorders are not directly related to its influence on corticosterone production. It is likely that MINO treatment in the ICVSTZ model, in addition to inhibiting neuroinflammation, also has a direct anti-inflammatory effect on the periphery associated with the suppression of STZ-induced overexpression of pro-inflammatory cytokines and the restoration of anti-inflammatory cytokines. Anti-inflammatory cytokines, such as IL-10, can inhibit neuroinflammation and promote neuroregeneration [7]. However, it should be emphasized that the anti-inflammatory effect induced by MINO therapy also includes a reduction in the number of TCD8^+^ lymphocytes observed in our study, which may be associated with a decrease in their cytotoxic activity and immune suppression. Furthermore, the anti-inflammatory action of MINO, which affects both neuroinflammation associated with sAD and inflammation in peripheral blood, may be related to its protective effect on blood–brain barrier (BBB) function. BBB is susceptible to hyperglycemia and chronic inflammation, which are early disorders observed during sAD after ICVSTZ injection and lead to brain insulin resistance [37,38,39]. Given the impact of the pathological response induced by hyperglycemia and neuroinflammation on BBB integrity and the critical role of BBB integrity on cognitive function [87,88], it is also likely that the neuroprotective effect of MINO through reducing inflammation results from stabilizing BBB integrity. Brown et al. [89] found that MINO reduces inflammation and protects BBB stability in small cerebral vessels. Furthermore, MINO inhibited the penetration and infiltration of peripheral AD monocytes crossing the BBB [90].

A limitation of our study is small group sizes for some biochemical measurements (Western blots) and that only a single dose of MINO (35 mg/kg b.w.) was used, whereas a dose–response study and longer treatment duration would be more informative to clinical utility. Furthermore, additional experiments (e.g., pharmacological inhibition of IDO1, APP knockdown, amyloid-β (Aβ) plaque deposition, kynurenine metabolites measurements or impact of gender) would clarify the mechanistic pathways of these effects. It may be the subject of future research.

## 4. Materials and Methods

### 4.1. Animals

**Figure 10:**
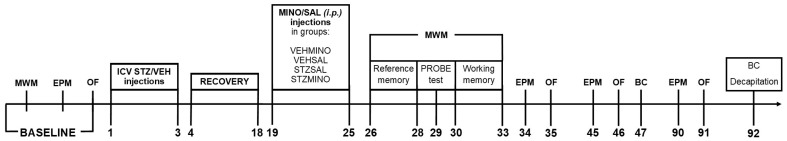
Diagram of the experimental procedure. Explanations: MWM—Morris water maze test (reference memory: 1–3 consecutive days of the MWM, probe test: day 4 of the MWM when the platform was eliminated, working memory: throughout the trials 1–4 of 5–8 consecutive days of the MWM); EPM—elevated plus maze; OF—white and light illuminated open field test; Baseline—prior to cannula implantation and injections; ICV STZ/VEH injections—intracerebroventricular (ICV) injections of streptozotocin (STZ, cumulative dose 3 mg/kg, split into two administrations on days 1 and 3, 0.75 mg/kg/2 µL/ ventricle, sporadic Alzheimer’s disease model induction); ICVVEH—intracerebroventricular (ICV) injections of citrate buffer (VEH, 2 µL/ventricle), Recovery—two-week period after stereotactic cannula implantation; MINO/SAL (i.p.) injections—intraperitoneal (i.p.) injection of minocycline (MINO) or saline (SAL) for 7 consecutive days; VEHMINO—ICV-injected rats with citrate buffer and i.p. injections of MINO; VEHSAL—ICV-injected rats with citrate buffer and i.p. injections of SAL; STZSAL—ICV-injected rats with STZ and i.p. injections of SAL; STZMINO—ICV-injected rats with STZ and i.p. injections of MINO; BC-blood collection; Decapitation of animals.

Experiments involving animals were conducted at University of Gdańsk, Faculty of Biology license number: 0169 in line with the Directive 2010/63/EU of the European Parliament, and on the basis of the authorization of the Local Ethical Committee for the Care and Use of Laboratory Animals at University of Technology in Bydgoszcz, Poland (No. 8/2019). A total of 40 male Wistar Han rats were acquired from a licensed breeding center (Tri-City Central Animal Laboratory, Research and Service Centre of the Medical University of Gdansk, Poland, breeder registration number 041). The acclimatization period in the animal facility lasted 14 days. The rats were then handled for 14 consecutive days, one rat about 5 min each day, to get them used to the experimenter and minimize stress during the experimental procedure. During the procedure, the rats were accustomed to a grip that exposed the peritoneum in order to minimize stress during intraperitoneal (i.p.) injections. Over the course of the experiment, the rats were kept separately in polycarbonate cages (width 20 cm, length 40 cm, height 18 cm) in a 12 h light/dark cycle (lights on at 06:00) in an air-conditioned room at a stable temperature (22 ± 2 °C), with water and food provided ad libitum. The animals could visually watch other individuals and were indirectly exposed to odors from other individuals’ cages. Prior to commencing behavioral testing under baseline conditions (prior to injections and cannula implantation), the rats reached a body weight of 300 ± 10 g, which was equivalent to 11–12 weeks of life. After finishing the basic behavioral surveys, the animals were randomly divided into four groups (Table 1): STZSAL (red is the group colour in all figures and tables) subjected to intracerebroventricular injections of streptozotocin (STZ) and intraperitoneal injections of saline (SAL); STZMINO (orange is the group colour in all figures and tables) subjected to intracerebroventricular injections of STZ and intraperitoneal injections of minocycline (MINO); control VEHSAL (green is the group colour in all figures and tables) subjected to intracerebroventricular injection of citrate buffer (VEH) and intraperitoneal injections of SAL; and control VEHMINO (light green is the group colour in all figures and tables) subjected to intracerebroventricular injection of VEH and intraperitoneal injections of MINO. The rats were then subjected to the experimental procedure according to the scheme shown in Figure 10.

### 4.2. Behavior in the Morris Water Maze (MWM)

Morris water maze (MWM) tests were conducted at the beginning of the study (before injections and stereotactic implantation, baseline) and then from 26 to 33 days after sAD induction (Figure 10). First, reference memory was measured (1–3 days of the MWM and probe test) and then working memory measurements (5–8 days of the MWM) according to the method that we described previously [91,92,93,94] with some modifications. A probe test for reference memory was conducted on day 29 after sAD induction (day 4 of the MWM, with one trial and with platform removed). The reference memory testing (3 days, four trials per day) was carried out with a platform that remains in a fixed position during all training sessions and working memory was performed with the location of the platform being changed every day (four trials a day for four days). In order to minimize experimenter bias, the behavioral activity of the rats in the MWM were saved using a video camera (EthoVision XT, Noldus, Wageningen, The Netherlands). During the probe test when the reference memory was tested, two parameters labeled in the MWM were measured: latency to reach a place where the platform previously was and percentage of time spent in the critical quadrant of the pool where the platform was situated during MWM days 1–3. Next, behavioral activity in the MWM was assessed by a trained observer who was unaware of the animals’ assignment to experimental groups.

### 4.3. Behavior in the Elevated Plus Maze Test (EPM)

Behavioral activity related to anxiety in the elevated plus maze (EPM) was conducted in accordance with the procedure we described earlier [92,93,95] with some modifications. Behavior in the EPM test was assessed between 8:00 and 12:00 a.m. The rats were always put in the maze by facing the open end of the maze and were left to explore the EPM for 5 min. After the test session, each rat was moved back to its own cage, and the whole apparatus was cleaned with a 70% ethanol solution and allowed to dry for 5 min to prevent any impact from odor signals. The recording was made at baseline (before injections and stereotactic cannulae implantation) and at days 34, 45, and 90 after ICVSTZ/VEH injections (Figure 10). Regarding the blinding, each observation session was recorded by a video camera (EthoVision XT, Noldus, Wageningen, The Netherlands). Registered reactions included the following: number of entries to the closed/open arms and center, and time in the closed/open arms and center of the maze. Behavioral activity in the EPM was assessed by a trained observer who was blind to the experimental procedure.

### 4.4. Behavior in the White and Illuminated Open Field (OF) Test

Anxiety-like behavior in the white and illuminated open field (OF) test was performed at baseline (prior to injections and the cannulas implantation) and on days 45 and 90 after ICVSTZ injection (Figure 10) in accordance with the procedure described earlier [70,71,72,92,96]. Because a lit and open chamber is more stressful for rodents than a dark, closed chamber [93], a 200 W white light lamp was placed 75 cm above the center of the open arena during the tests. The test rats were put out in one corner of the area and allowed to remain in the test room for 30 min. Following the time of the exposure to OF, the arena was cleaned with water, alcohol, and then again with water. To minimize researcher bias, the rat’s behavioral activity in the open field arena was recorded by a video camera (EthoVision XT, Noldus, Wageningen, The Netherlands). Behavioral activity recorded during each 30 min observation session included the following: exploration (measured as number of lines crossed), freezing time, time at periphery and center, center entries, rearing, grooming, frequency of defecation, and miction.

### 4.5. Intracerebroventricular (ICV) Injections of Streptozotocin (STZ)—A Model of Sporadic Alzheimer’s Disease (sAD)

To induce the rat sAD model, ICV injections of streptozotocin (STZ) or vehicle (VEH: 0.02 M citrate buffer, pH 4.5) were carried out in accordance with the method described above [91,92,93,94] with some modifications. In summary, rats anesthetized with 2.5% isoflurane (air flow: 0.5 L per minute) were implanted with the cannulas (22GA, 9 mm long, Plastic One, Roanoke, VA, USA) with a stereotactic apparatus (Hugo Sachs Elektronik, Germany) into lateral ventricles (coordinates: AP: −1.3 mm, L: ±2 mm, D: +3.6 mm according to bregma) [97]. ICV infusions of STZ at a cumulative dose of 3 mg/kg were administered in duplicate at a rate of 1 μL/min on days 1 and 3 (2 × 1.5 mg/kg, dissolved in citrate buffer 0.02 M, pH 4.5; 0.75 mg/kg dissolved in 2 μL of vehicle (citrate buffer) for each lateral ventricle). Control rats were subjected to ICVVEH injections according to the same procedure as ICVSTZ administration. The drug injections were made by means of a microinfusion pump (Legato-100—Series Syringe Pump, KD SCIENTIFIC, Holliston, MA, USA), and a Hamilton syringe (10 μL) connected to an injection cannula (28GA, 11 mm long, Plastic One, Roanoke, VA, USA) was placed into the guide cannula 2 mm below its tip. To prevent fluid from flowing out, the injection cannula was kept in the guide for an extra 60 s. As soon as the stereotactic cannulas were implanted, the rats were taken to a warm room, where they stayed until they became conscious again. The rats were then allowed a convalescence period of two weeks to heal wounds.

### 4.6. Minocycline (MINO) Treatment

Minocycline (MINO) (#M9511, Sigma-Aldrich, St. Luis, MO, USA) was administered intraperitoneally (i.p.) at a dose of 35 µg/kg b.w., once daily for 7 consecutive days (from day 19 to 26 after ICVSTZ/VEH injection, Figure 10). The dose of MINO, which was used in rats in our studies, was chosen based on the literature [30] and was effective in improving behavioral activity related to reference memory, which manifested itself in shorter latency to reach the platform on days 1–3 of the MWM test (trial 1). Other authors have shown that a similar dose of MINO (30 mg/kg b.w.) administered for 5 consecutive days (i.p.) was sufficient to reduce microglia recruitment and the morphological profile of inflammation while restoring learning and memory abilities impaired by ICVSTZ (2 mg/kg) injection [53]. Furthermore, MINO at a dose of 20 or 40 mg/kg injected (i.p.) for 7 consecutive days had a neuroprotective effect on memory deficits caused by ischemia/reperfusion [33,34].

### 4.7. Measurement of Interleukin (IL)-6, IL-10, and Corticosterone Concentrations in Plasma and TCD4+/TCD8+ Lymphocyte Number in Blood

Blood samples were collected from the hearts of rats under anesthesia using isoflurane (2.5%, air flow: 0.5 L per minute) pump (Bitmos OXY 6000, Bitmos GmbH, Dusseldorf, Germany) at days 47 and 92 after ICVSTZ/SAL injection. On day 92 after sAD induction (one day after the last behavioral session), rats under isoflurane anesthesia were euthanized with Morbital (2 mL/kg); blood samples and brains were taken from all rats.

#### 4.7.1. Determination of Plasma Pro-Inflammatory Interleukin (IL)-6 and Antiinflammatory IL-10 Concentration and Peripheral Blood Mononuclear Cells (PBMC)-Derived Production of IL-6 and IL-10

Concentration of plasma IL-6 and IL-10 and their production in blood were determined according to the method we described previously [70,71,72,91]. Briefly, peripheral blood mononuclear cells (PBMC) were separated from heparinized blood by Ficoll 400 (Pharmacia, Uppsala, Sweden) and the Uropolinum (Polfa, Starogard, Poland) density centrifugation method (1113× *g*, 30 min at 4 °C). The isolated cells were washed with phosphate-buffered saline. PBMC suspension in RPMI-1640 with a 10% calf bovine serum were seeded at a concentration of 4 × 10^6^ cells/mL and used in concanavalin-A (Con-A)-stimulated production of pro-inflammatory IL-6 and anti-inflammatory IL-10. PBMC suspensions in complete medium were put into 24-well Corning tissue culture plates and subsequently stimulated with Con A solution (2.5 g/mL) or left unstimulated (control samples). This mixture of PBMC and Con-A solution was incubated at 37 °C and with a 5% CO_2_ flow. Cell-free supernatants were harvested after 48 h of incubation and stored at −20 °C up to the time of analysis. Cell vitality was evaluated with Trypan Blue.

IL-6 and IL-10 plasma and supernatant concentrations were measured by ELISA using Rat-IL-6 and Rat-IL-10 ELISA kits (Invitrogen, ERA31RB and ERA23RB, Waltham, MA, USA). Briefly, standards or samples were administered to 96 wells precoated with rat IL-6 and IL-10 antibody, as appropriate, and then incubated for 2.5 h (IL-6) and 1 h (IL-10) at ambient temperature. After thorough rinsing with wash buffer, 100 μL of the biotinylated anti-IL-6 or anti-IL-10 were given to every well and the dishes were incubated over 1 h at ambient temperature. When rinsed, 100 μL of streptavidin-HRP was incorporated, and the samples were incubated for 45 min and repeatedly rinsed. Next, 100 microliters of tetramethylbenzidine was applied to each well. On the completion of 10 min, the reaction was stopped and the absorbance quantified with a DTX 880 Multimode Detector system (Beckman Coulter, Brea, CA, USA) fixed at 450 nm reaction. Concentration of cytokines were determined from a standard curve produced by Beckman Coulter’s Biomek software version i5 derived from the absorbance of standard samples. The detection sensitivity measured was 16 pg/mL for IL-6 and 3 pg/mL for IL-10.

#### 4.7.2. Flow Cytometry Analysis of T Helper (CD3^+^CD4^+^) and T Cytotoxic (CD3^+^CD8^+^) Lymphocyte Subpopulations

PBMC adjusted to 1 × 10^7^ cells/mL in complete medium were used in the flow cytometry analysis of TCD3^+^CD4^+^ (T helper) and TCD3^+^CD8^+^ (T cytotoxic) lymphocytes. Flow cytometry analysis was carried out in accordance with the methodology we previously described [70,91,92,93,95] with some modifications. PBMC suspension (25 µL) was mixed with 25 µL of IOTest CD3-FITC/CD4-PC7/CD8-APC (Beckman Coulter, A07800, Brea, CA, USA). After mixing, the samples were incubated at ambient temperature over 20 min in the dark, fixed and stored at 4 °C up to the time of flow cytometry performed using Cytomics FC 500 flow cytometer (Beckman Coulter, Brea, CA, USA) and MXP Software version 2.1. The percentage of T lymphocyte subpopulations was evaluated, gaiting on forward and side scatter characteristics.

### 4.8. Plasma Corticosterone Measurement

At days 47 and 92 after ICVSTZ/SAL injection, blood samples from rats under anesthesia (2.5% isoflurane, airflow: 0.5 L/min) were harvested through a cardiac puncture (9–10 a.m.) and plasma corticosterone concentrations were tested in two repetitions using a radioimmunoassay method (rCorticosterone (125I) RIA KIT, RK-548, isotop Institute of Isotopes Co, Ltd., Budapest, Hungary, sensitivity: 0.5 ng/mL), in accordance with the method we described earlier [70,71,72,91,92,93] using Wizard 1470 gamma counter (Pharmacia—LKB, Turku, Finland). The lowest detection dose with this assay system was 7.7 ng/mL.

### 4.9. Isolation of Brain Structures

Ninety-two days after sAD induction or VEH injection, rats under anesthesia with 2.5% isoflurane (air flow: 0.5 L per minute) were euthanized: some rats randomly selected from each group were killed by decapitation (Figure 10), and the remaining rats underwent transcardial perfusion first with saline, then with paraformaldehyde, and the isolated and fixed brains were frozen until further immunohistochemical procedures. The prefrontal cortex and hippocampus were then isolated from the brains after decapitation and subjected to biochemical analysis of APP and IDO1 by Western blot. Immediately after decapitation and brain collection, the brain was placed on a glass plate with ice, and the prefrontal cortex and hippocampus were isolated. The isolated structures were frozen at—70 degrees until further analysis.

### 4.10. Reagents and Antibodies for the Measurement of IDO1 and APP Levels in the Brain

APP and IDO1 levels were measured in isolated structures from brains after decapitation: the hippocampus and prefrontal cortex. Bovine serum albumin (BSA) was obtained from Merck (Darmstadt, Germany). The rabbit monoclonal anti-IDO antibody was purchased from ABclonal Technology (Woburn, MA, USA), whereas the rabbit monoclonal anti-APP was obtained from OriGene (Rockville, MD, USA). The monoclonal anti-β-actin-peroxidase and the secondary anti-rabbit HRP antibodies were from Merck.

### 4.11. Sample Preparation and Western Blotting

Protein samples from frozen brain structures were extracted in RIPA buffer (Eurx, Gdańsk, Poland) supplemented with protease inhibitor mixture (Roche Life Sciences, Basel, Switzerland) using the Tissue Lyser II (Qiagen, Hilden, Germany) homogenizer. The homogenates containing proteins were sonicated (5 min, 40% output) and centrifuged (5000× *g*, 5 min, 4 °C). Protein concentration was measured by Qubit Protein Assay (Thermo Fisher Scientific, Waltham, MA, USA), using Qubit 2.0 Fluorometer (Thermo Fisher Scientific).

Samples were resolved by reducing SDS/PAGE (12% gels). The proteins were transferred onto Immobilon-FL membrane (Merck) by Trans-Blot Turbo Transfer System (Bio-Rad, Hercules, CA, USA). Membranes were then blocked with 5% milk or 3% BSA (for IDO1 detection) and incubated with the appropriate primary and secondary antibodies, diluted in 5% milk or 3% BSA for anti-IDO1. Proteins were detected by chemiluminescence with Clarity Max ECL Western Blotting Substrate (Bio-Rad) and visualized using Azure Imager c400 (Azure Biosystems, Dublin, CA, USA). Signal intensities of the bands were quantified using Image Studio Lite (v.5.2) (LI-COR Bioscences, Lincoln, NE, USA).

### 4.12. Data Analysis

Statistica 13 PL (Statsoft Polska Sp. z.o.o., Kraków, Poland) was used for a statistical analysis of the results. The normality of the variables’ distribution was verified using the Shapiro–Wilk test and the homogeneity of the variances with a Levene test. As the result of the Shapiro–Wilk test indicated that the assumptions of the parametric analysis were not met, we used this to further statistically evaluate the differences in behavioral activity, peripheral immunity, and endocrine variables using non-parametric statistical tests. The Kruskal–Wallis ANOVA by ranks for multiple comparisons (time: baseline and ICVSTZ post-injection days; treatment: STZ, MINO, VEH, SAL) was used for the effect on behavior and peripheral inflammatory markers. A comparison of the groups was made using the Mann–Whitney U test. Statistical analyses for the hippocampal and prefrontal cortex APP and IDO1 levels were performed by one-way ANOVA followed by Dunnett’s test. All experiments for brain APP and IDO1 level measurements were made independently at least three times. All values are expressed as mean ± SD. A *p* value lower than 0.05 was considered statistically significant.

## 5. Conclusions

Seven-day MINO treatment (35 mg/kg b.w., i.p.) in the rat model of sAD protects against ICVSTZ injection-induced early reference memory deficits related to sAD anxiety-like behavior in the late stage of disease progression. The MINO-induced improvement in spatial memory and anxiety behavior was accompanied by the downregulation of such inflammatory markers as IDO1 and decreased APP levels in the hippocampus and prefrontal cortex with simultaneous stimulation of the peripheral anti-inflammatory response, as indicated by increased production of anti-inflammatory IL-10 in the blood. Our results highlight that inflammation plays a crucial role in the early stages of sAD, involving the modulation of IDO1 and APP expression. This suggests that the antibiotic MINO may be a potential therapeutic drug for use in the treatment of early, pre-plaque stages of sAD and associated anxiety behaviors, particularly due to its anti-inflammatory properties. The results also highlight that the approach to using MINO in psychiatric and neurodegenerative diseases draws on new knowledge linking its anti-inflammatory mechanisms to neuroprotective effects.

## Figures and Tables

**Table 1:** Experimental groups.

Group	Treatment	
VEHSAL *n* = 10	citrate buffer (VEH) icv	saline (SAL) i.p.
VEHMINO *n* = 10	citrate buffer (VEH) icv	minocycline (MINO) i.p.
STZSAL *n* = 10	streptozotocin (STZ) icv	saline (SAL) i.p.
STZMINO *n* = 10	streptozotocin (STZ) icv	minocycline (MINO) i.p.

## Data Availability

The original contributions presented in this study are included in the article and Supplementary Material. Further inquiries can be directed to the corresponding authors.

## Supplementary Materials

The following supporting information can be downloaded at: https://www.mdpi.com/article/10.3390/ijms26199397/s1.
