# Supplementary material for: Minocycline Treatment Improves Memory and Reduces Anxiety by Lowering Levels of Brain Amyloid Precursor Protein and Indoleamine 2,3-Dioxygenase in a Rat Model of Streptozotocin-Induced Alzheimer’s Disease
Source: Int J Mol Sci. 2025 Sep 26;26(19):9397. doi: 10.3390/ijms26199397 (PMC12524683; doi:10.3390/ijms26199397)

Figure 8. Western blot analysis of amyloid precursor protein (APP), indoleamine 2,3-dioxygenase 291 (IDO1), and actin in the hippocampus

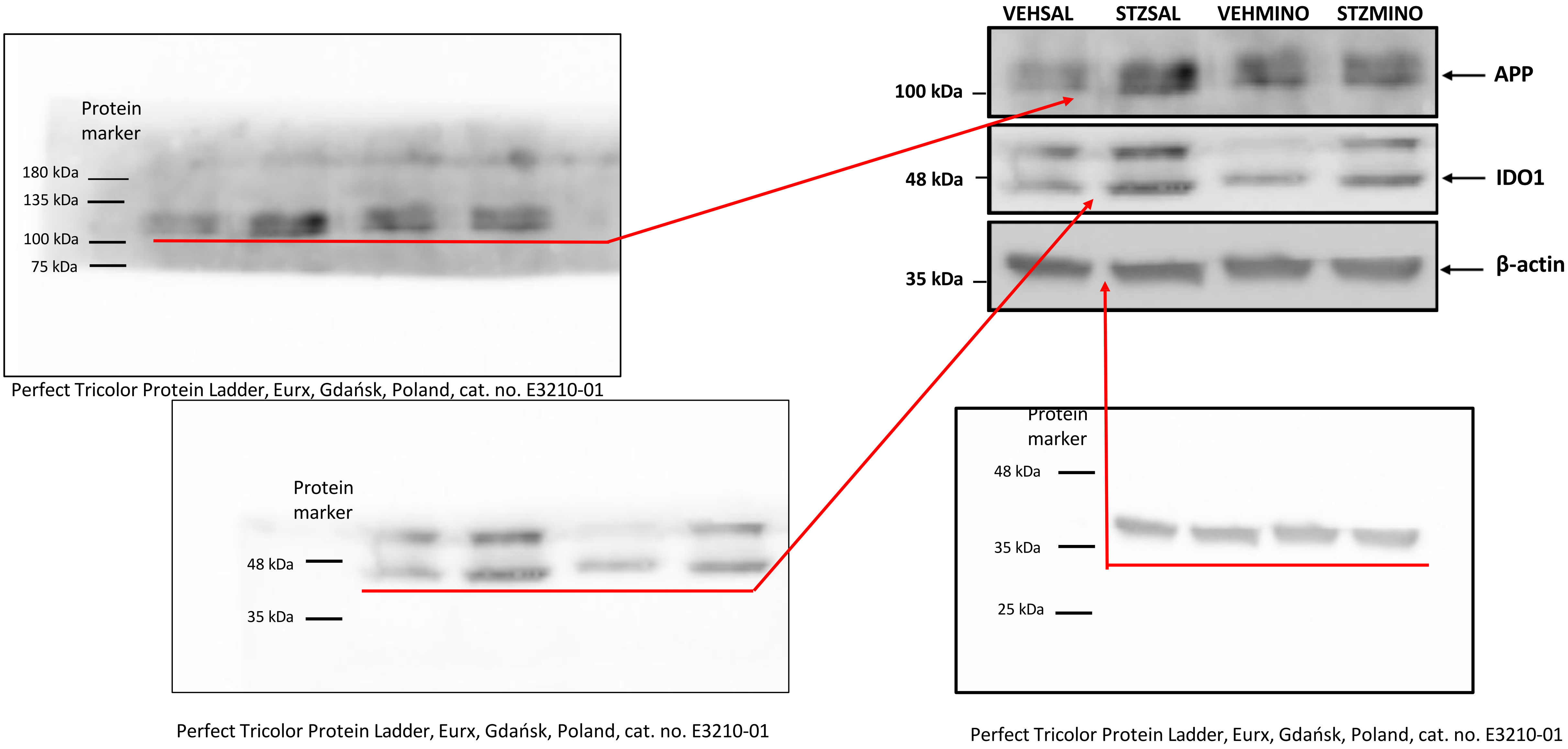

Supplement: Supplementary file 1 [file ijms-26-09397-s001.zip › ijms-3849616-supplementary/Figure S1 original images of Western blot/Figure 8_original images of Western blot.pdf]
