# Supplementary material for: Minocycline Treatment Improves Memory and Reduces Anxiety by Lowering Levels of Brain Amyloid Precursor Protein and Indoleamine 2,3-Dioxygenase in a Rat Model of Streptozotocin-Induced Alzheimer’s Disease
Source: Int J Mol Sci. 2025 Sep 26;26(19):9397. doi: 10.3390/ijms26199397 (PMC12524683; doi:10.3390/ijms26199397)
Supplement: Supplementary file 1 [file ijms-26-09397-s001.zip › ijms-3849616-supplementary/Table S1 Re-ijms-3849616.pdf]

Table S1. Concanavalin-A (Con-A)-stimulated interleukin (IL)-6 production by peripheral blood mononuclear cells (PBMC) in rats at 47 day after intracerebroventricular (ICV) injection of streptozotocin (STZ) or citrate buffer (VEH) and intraperitoneal (i.p.) injection of saline (SAL) for 7 consecutive days (STZSAL, n=10; VEHSAL, n=10) or ICV injection of STZ or VEH and i.p. minocycline (MINO) injection for 7 consecutive days (STZMINO, n=10; VEHMINO, n=10).

**Group**	**Control (without Con-A stimulation) mean ± SD [pg/ml]**	**Con-A stimulation mean ± SD [pg/ml]**
VEHSAL	180.5 ± 15.9	620.4 ± 12.5
VEHMINO	95.8 ± 9.1	120.0 ± 8.1
STZSAL	291.5 ± 17.5	455.3 ± 10.3
STZMINO	198.1 ± 14.8	207.4 ± 24.1
